# A decade of pollen transcriptomics

**DOI:** 10.1007/s00497-015-0261-7

**Published:** 2015-03-12

**Authors:** Nicholas Rutley, David Twell

**Affiliations:** Department of Biology, University of Leicester, Leicester, LE1 7RH UK

**Keywords:** Pollen, Male gametophyte, Transcriptome, Gene expression, Development

## Abstract

**Overview of pollen transcriptome studies.**

Pollen development is driven by gene expression, and knowledge of the molecular events underlying this process has undergone a quantum leap in the last decade through studies of the transcriptome. Here, we outline historical evidence for male haploid gene expression and review the wealth of pollen transcriptome data now available. Knowledge of the transcriptional capacity of pollen has progressed from genetic studies to the direct analysis of RNA and from gene-by-gene studies to analyses on a genomic scale. Microarray and/or RNA-seq data can now be accessed for all phases and cell types of developing pollen encompassing 10 different angiosperms. These growing resources have accelerated research and will undoubtedly inspire new directions and the application of system-based research into the mechanisms that govern the development, function and evolution of angiosperm pollen.

**Electronic supplementary material:**

The online version of this article (doi:10.1007/s00497-015-0261-7) contains supplementary material, which is available to authorized users.

## Introduction

**Fig. 1:**
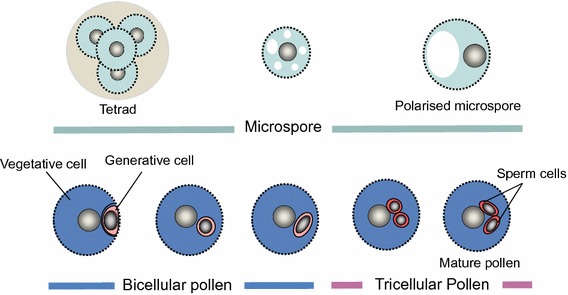
Distinct cytological stages that accompany male gametophyte development in Arabidopsis. Diploid pollen mother cells undergo meiotic division to produce a tetrad of haploid microspores. Microspores released from the tetrad undergo a highly asymmetric cell division (pollen mitosis I) to produce a bicellular pollen grain with a small generative (germline) cell within the cytoplasm of the larger vegetative cell. The generative cell undergoes a further mitotic division (pollen mitosis II) during pollen maturation to produce a pair of sperm cells

The haploid male gametophyte (or pollen) of flowering plants is a well-understood and intriguing cell system in which to study gene expression and its regulation, as its development involves single-cell ontogeny and the cooperation of two-cell lineages to enable double fertilisation (Twell 2011). Pollen develops from haploid microspores that are produced following meiosis within the anthers (Fig. 1). The four haploid microspores are initially associated in a tetrad, but typically separate and undergo vacuolation and expansion, with the microspore nucleus migrating towards the cell wall. In this polarised arrangement, pollen mitosis I (PMI) results in a bicellular pollen (BCP) grain composed of a generative cell (representing the male germline) enclosed within the vegetative cell cytoplasm. The vegetative cell exits the cell cycle, but the generative cell elongates and divides at pollen mitosis II (PMII), giving rise to a pair of sperm cells. Upon pollination of a receptive stigma, pollen grains hydrate and germinate to produce a pollen tube. The pollen tube grows through the pistil by tip extension, guided by sporophytic and female gametophyte-derived signals, to deliver the sperm cells into the ovule where double fertilisation takes place.

In this review, we provide a brief survey of historical advances in understanding of gene expression in the male gametophyte and review the scale and diversity of the transcriptome data that have accumulated in the past decade. Some allied topics of importance including meiosis, pollen proteome studies, and the emerging role of small RNAs are not covered in detail, and the reader is referred to other recent and topical reviews (Le Trionnaire and Twell 2010; Twell 2011; Baroux et al. 2011; Feng et al. 2013; Dukowic-Schulze and Chen 2014; Fu and Yang 2014; Kawashima and Berger 2014).

## Pollen gene expression in the pre-genomic era

**Fig. 2:**
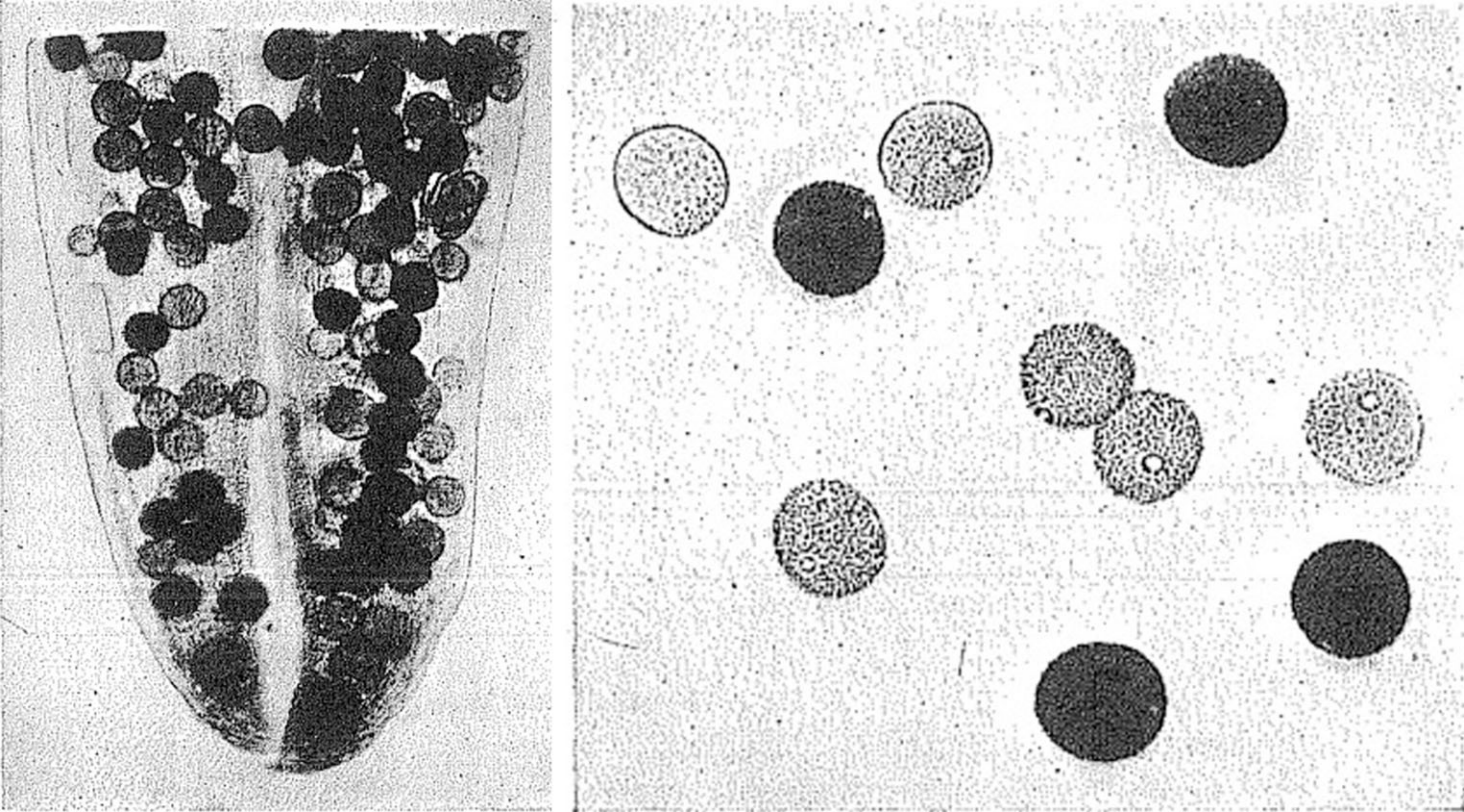
Images from an original plate by Parnell (1921) showing segregation of F1 pollen into *starchy* (*dark*) and *glutinous* (*light*) types. Anther (*left*) and free pollen (*right*). Parnell describes the result as follows: ‘With a view to distinguishing the two genetic types, the pollen was treated with iodine. The result was most satisfactory—two distinct types became evident, one giving the *dark blue* reaction of ordinary starch and the other the reddish reaction of amylodextrine’. With kind permission from Springer Science + Business Media

The first evidence for haploid gene expression in pollen can be traced back to the first half of the twentieth century. In 1921, Parnell observed that half of the pollen from rice plants, which were heterozygous for the recessive *glutinous* (or *waxy*) endosperm phenotype, stained reddish, rather than dark blue, with iodine (Fig. 2). The phenotypic segregation of the Waxy locus, which is responsible for amylose synthesis and encodes granule bound starch synthase I (Hirano and Sano 1991), thus provided genetic evidence for gene activity in pollen. Similar observations were made for *waxy* maize pollen and endosperm by Brink and MacGillivray (1924), who observed reduced pollen transmission of *waxy* alleles and hypothesised that this may be caused by reduced pollen tube growth, ‘by the action of certain factors active in the tube nucleus’. Male-biased and distorted Mendelian segregation ratios have been detected repeatedly and include examples where mutant alleles affect pollen development, germination, or pollen tube growth (reviewed in Ottaviano and Mulcahy 1989; Twell 1994).

More recently, the directed isolation of developmental mutants and their associated genes has provided insight into the spatiotemporal expression of important genes in the male gametophyte. An innovation in the search for such genes was the use of the fluorescent DNA stain 4′,6-diamidino-2-phenylindole (DAPI) to screen for abnormal pollen in mutagenised populations of Arabidopsis by fluorescence microscopy. This approach was used to identify the LOB domain family protein SIDECAR POLLEN (SCP), required for correct timing and organisation of PMI (Chen and McCormick 1996; Oh et al. 2010); the MAP215–family GEMINI POLLEN1 (GEM1), required for microspore polarity and asymmetric division at PMI (Park et al. 1998; Twell et al. 2002), as well as the TWO-IN-ONE (TIO) fused kinase required for pollen cytokinesis (Oh et al. 2005, 2010) and the germline-specific transcription factor DUO POLLEN1 (DUO1) (Durbarry et al. 2005; Rotman et al. 2005). A second strategy made use of T-DNA or transposon-derived resistance markers in segregation distortion screens for deviations in the ratio of resistant to sensitive progeny from 3:1 to ≤1:1. They delivered developmental mutants, such as *limpet pollen* in which the germ cells remain attached to the pollen wall (Howden et al. 1998), and progamic phase mutants affecting pollen tube growth and/or guidance (Procissi et al. 2001; Lalanne et al. 2004; Johnson et al. 2004). Arabidopsis mutants affecting all stages of pollen development, from the development of the microspore to the pollen tube as well as male–female gamete interactions, have now been isolated (reviewed by Twell 2011; Mori et al. 2014).

While segregating pollen mutants provided compelling evidence for developmentally regulated haploid gene expression, the first steps towards describing the RNA landscape of pollen began with the characterisation of different classes of RNA. Studies in *Tradescantia*, *Lilium,* and tobacco showed the rate of accumulation of rRNA and tRNA to be dynamic and variable between species (see Mascarenhas 1990). In *Tradescantia* and *Lilium*, rRNA and tRNA levels peaked around PMI, while in tobacco, levels of rRNA, tRNA, and mRNA increased progressively after PMI (Schrauwen et al. 1990). The detection of large amounts of mRNA in mature pollen further suggested that transcripts are stored for use during pollen germination and tube growth.

Initial studies to explore the composition of mRNA populations involved in vitro translation of mRNA from developing pollen of tobacco and *Lilium* (Schrauwen et al. 1990). For both species, the resulting 2D protein profiles revealed the presence of different transcripts before and after PMI, with the greatest number of new transcripts appearing in mature pollen. These and similar findings in maize added weight to the evidence for post-meiotic transcription, based on the accumulation profiles of cloned pollen-expressed and pollen-specific transcripts, such as actin and the familiar tomato late pollen gene *LAT52*, respectively (Stinson et al. 1987; Twell et al. 1989). Two major groups of genes were recognised: ‘early’ genes first expressed in the microspore, which decreased in abundance before pollen maturation, and ‘late’ genes expressed after PMI, which accumulated until maturity, although more complex patterns were apparent (reviewed by Mascarenhas 1990, 1992; Twell 1994, 2002). A further level of cell-specific control of transcription was recognised when the tomato *LAT52* promoter was linked to a nuclear-targeted GUS fusion protein gene, which revealed that the *LAT52* promoter was active in the vegetative nucleus, but not in the generative cell (Twell 1992).

**Fig. 3:**
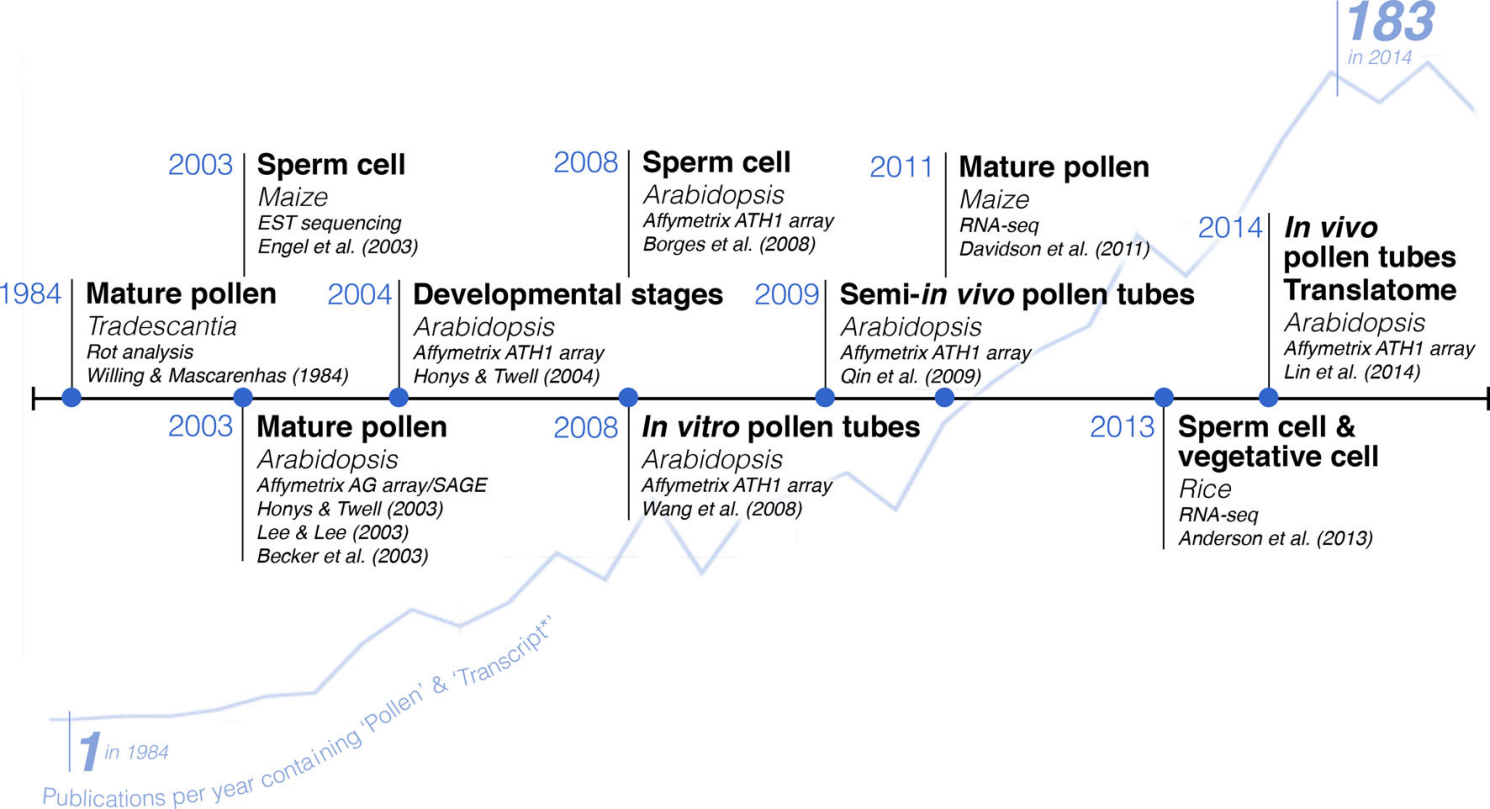
A timeline charting some of the important advances in male gametophyte transcriptome studies. The developmental stage, cell type, technique, species, and associated publications are indicated. The remarkable increase in the rate of published work associated with the terms ‘pollen’ and ‘transcript*’ is illustrated by the *line graph* indicating the numbers of publications per year (Web of Science™ version 5.16)

The first predictions of pollen transcriptome size were based on poly(A)RNA–cDNA association kinetic (R_0_t) analysis, from which maize and *Tradescantia* pollen were estimated to express around 20,000 different transcripts, representing around 30 % fewer than in shoots (Willing and Mascarenhas 1984; Willing et al. 1988). It is only in the past decade, however, that genomic platforms have been used to validate these estimates and to explore the full repertoire of genes expressed throughout the different phases of pollen development. Some of the important advances are charted in Fig. 3, together with the remarkable increase in the annual rate of publications reporting on pollen and transcription.

## Pollen gene expression in the post-genomic era

By the turn of this century, gene-by-gene studies had identified around 150 different pollen-expressed genes associated with a range of functions, including cell wall metabolism, cytoskeleton, transcription, and signalling (see Twell 2002). The plants investigated were numerous (>28 species) and diverse contrasting with the studies of the subsequent decade, which initially were focussed on the model *Arabidopsis thaliana.* The obvious limitations of gene-by-gene studies were overcome with advances in transcriptome profiling, which included serial analysis of gene expression (SAGE) and microarray technology, allowing users to simultaneously monitor the expression of thousands of genes. For example, the first-generation Affymetrix Arabidopsis AG array or 8-K GeneChip harboured probe sets for around 8100 genes, approximately one-third of the Arabidopsis gene models known from EST collections and cDNA libraries (Richmond and Somerville 2000).

The transcriptome of mature pollen was first investigated in three studies, which provided a coherent overview of pollen gene expression, two utilising 8-K microarrays (Honys and Twell 2003; Becker et al. 2003) and the third SAGE technology (Lee and Lee 2003). The microarray studies led to the identification of 992 (Honys and Twell 2003) and 1587 (Becker et al. 2003) genes expressed in mature pollen estimating the total number of pollen-expressed genes in Arabidopsis between 3500 and 5500. The pollen transcriptome showed a unique profile and was enriched for genes involved in signalling, cell wall metabolism, and the cytoskeleton, but under-represented for energy metabolism, transport, transcription, and translation. The presence of transcripts for a large number of cell wall-associated proteins (e.g. glycoside hydrolases, polygalacturonases, and cellulases) and cytoskeletal components (e.g. actin and profilin), highlighted a significant role for storage and translation of mRNAs after pollination, where pollen germination and tube growth require significant modifications to the cell wall and dynamic regulation of the actin cytoskeleton (Honys and Twell 2003). The enrichment of signalling components in the pollen transcriptome was also in accordance with the important role of pollen–pistil interactions and female gametophytic signals, which support and guide the pollen tube to the ovule (reviewed by Palanivelu and Tsukamoto 2012).

With the release of the Arabidopsis genome, the second-generation ATH1 Genome Array (Affymetrix) was developed, harbouring probe sets for around 24,000 genes (Redman et al. 2004). However, around 3000 genes had no representation on the ATH1 platform, including important regulators, such as the male germline-specific transcription factor DUO1 (Rotman et al. 2005; Brownfield et al. 2009a, b). The ATH1 platform represented a major advance and a substantial body of data now exists for sporophyte-free Arabidopsis pollen, with >130 raw data files available in Gene Expression Omnibus and ArrayExpress (Tables S1 and S2). These data have not only provided the essential raw materials required to decipher the contribution of individual genes and gene families to male gametophyte development, but also guided the design of reverse genetics approaches and the analysis of gametophytic pollen mutants (Table S1). For example, pollen developmental transcriptome data were initially exploited for the analysis of cation/H^+^ exchanger proteins (Sze et al. 2004) and more widely for all putative transporters (Bock et al. 2006), enabling functionally redundant proteins, such as CHX21 and CHX23, required for pollen tube guidance to the ovules to be identified (Lu et al. 2011).

Given that near-comprehensive transcriptome data sets have been generated in Arabidopsis for different phases of pollen development, including the germline and progamic phase (Table 1), we review these to highlight the major advances, followed by a discussion of the impact of comparative pollen transcriptome studies (see previous reviews by Twell et al. 2006; Becker and Feijo 2007; Schmidt et al. 2012).

**Table 1:** A summary of male gametophyte stage-specific transcriptome studies for different plant species

	UNM	BCP	TCP	MPG	PT	GC	SC	VC
*A. thaliana*	X	X	X	X	X		X	
*O. sativa*	X	X	X	X	X		X	X
*N. tabacum*	X	X	X^1^	X	X		X^EST^	
*Z. mays*				X			X^EST^	
*L. longiflorum*				X	X	X^EST^		
*G. max*				X				
*V. vinifera*				X				
*F. vesca*				X				
*S. lycopersicum*				X				
*P. zeylanica*							X^EST^	

Developmental stages: UNM, microspore; BCP, bicellular pollen; TCP, tricellular pollen; MPG, mature pollen; PT, pollen tube; GC, generative cell; SC, sperm cell; VC, vegetative cell. X, signifies at least one microarray or RNA-seq data set. X^EST^, EST data is published for *N. tabacum* (Xin et al. 2011) and *Z. mays* (Engel et al. 2003) sperm cells and *L. longiflorum* generative cells (Okada et al. 2006). X^1^, generative cell division occurs during pollen tube growth in *N. tabacum*. See Table S1 and Table S2 for additional references

## The Arabidopsis pollen transcriptome

**Fig. 4:**
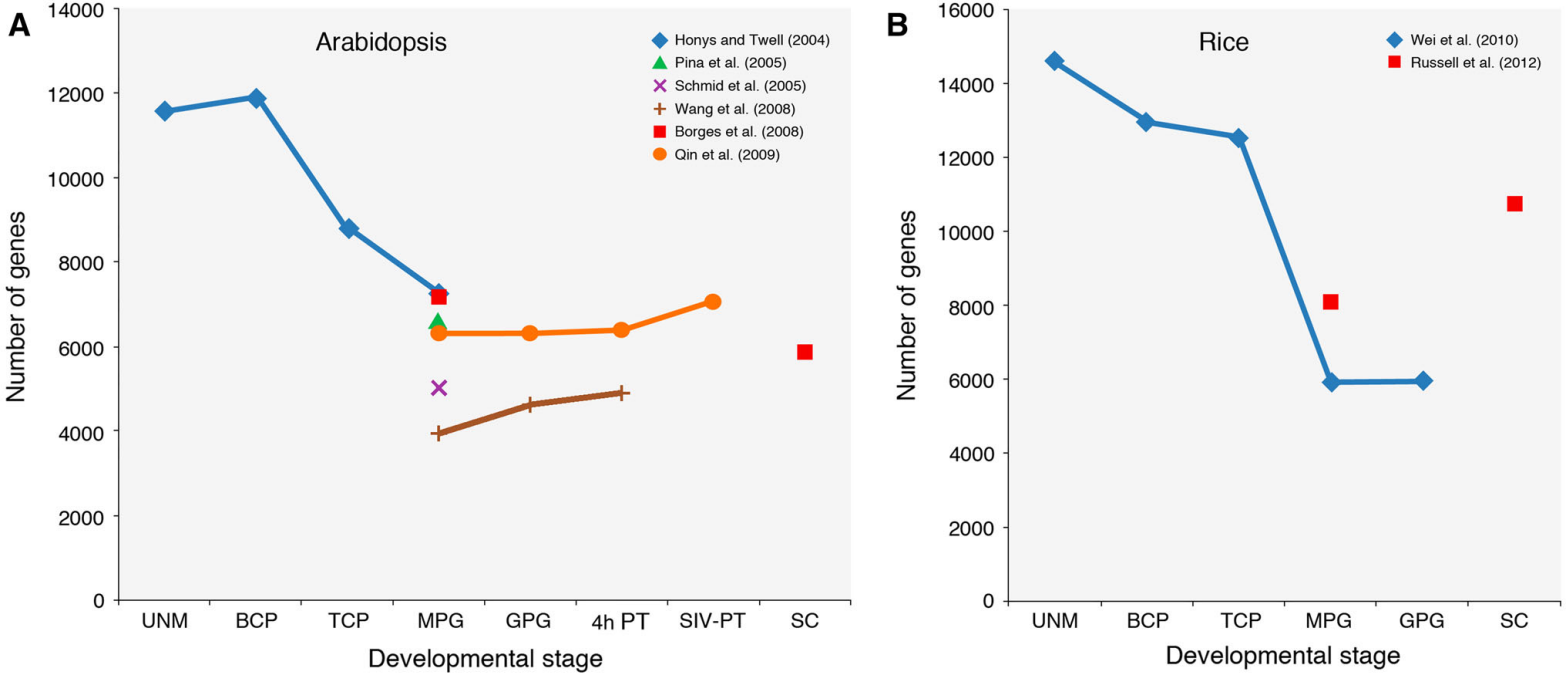
Plots of transcriptome sizes at different stages of male gametophyte development based on studies using Arabidopsis ATH1 (**a**) or rice 57 K (**b**) microarrays. UNM, uninucleate microspores; BCP, bicellular pollen; TCP, tricellular pollen; MPG, mature pollen; GPG, germinating pollen; 4-h PT, 4-h in vitro-grown pollen tubes, SIV-PT, semi-in vivo pollen tubes. SC, purified sperm cells

Several groups have provided independent estimates of the number of genes expressed in mature pollen, which range from 3954 to 7235 genes, with an average of 6044 genes (Honys and Twell 2004; Pina et al. 2005; Schmid et al. 2005; Borges et al. 2008; Wang et al. 2008; Qin et al. 2009–see Fig. 4a). The use of different Arabidopsis accessions, pollen states (desiccated or hydrated), sample collection methods, and detection call algorithms may have contributed to the large variation in these estimates. Similarly, estimates of the percentage of genes that are pollen-specific also vary, from 4 to 11 %, depending on the normalisation algorithms used and the number and diversity of sporophytic data sets used for comparison (Boavida et al. 2005; Twell et al. 2006). There are however two major findings common to all studies: first, the unique composition of the pollen transcriptome; second, its striking reduction in complexity compared with sporophytic tissues and purified sporophytic cell types, such as root hair cells (11,696 genes; Becker et al. 2014) and stomatal guard cells (13,222 genes; Bates et al. 2012).

RNA-seq is a powerful alternative approach to study the pollen transcriptome, with higher sensitivity and a broader dynamic range (Zhao et al. 2014), which overcomes the limitations of promiscuous probe sets and gene models not represented on the ATH1 microarray. In the first RNA-seq analysis of Arabidopsis pollen, Loraine et al. (2013) reported at least 4172 protein-coding genes to be expressed. Although the number of genes detected was lower than estimates based on the majority of ATH1 studies, a highly conservative threshold of five reads per million (RPM) was applied, and reads mapping to multiple locations in the genome assembly were discarded. Reprocessed data now available in the online version of Loraine et al. (2013) provide normalised data as reads per kilobase per million (RPKM). In pollen, 6722 genes were expressed at 1 RPKM or above, including 6473 protein-coding genes, which is greater than the average number of pollen-expressed genes (6044) detected using ATH1 arrays. Illustrating the detection sensitivity of this threshold, we found that transcripts for two well-known male germline-specific genes, *DUO1* and *GCS1/HAP2,* were absent from seedlings but present in pollen at 2.4 and 3.0 RPKM, respectively. Nevertheless, the overlap between pollen ATH1 and RNA-seq data was almost complete, with <1 % of genes reliably detected on the ATH1 array not being found in RNA-seq data. Interestingly, 11 % of the genes detected by RNA-seq had no corresponding probe sets on the ATH1 array, revealing a previously unknown group of pollen-expressed genes. Pollen-expressed genes with additional exons and genes with previously unannotated 5′ and 3′ untranslated regions were also identified, although fewer than 20 genes annotated as alternatively spliced in TAIR10 were differentially spliced between pollen and seedlings. The RNA-seq data set of Loraine et al. (2013) thus provides a useful resource for the evaluation of annotation accuracy, alternative splicing, and a point of reference for future RNA-seq-based studies of Arabidopsis pollen gene expression.

## The Arabidopsis male germline transcriptome

The male germline makes a unique contribution to the pollen transcriptome (Engel et al. 2003). This was first revealed in Arabidopsis by profiling RNA from fluorescence-activated cell sorting (FACS)-purified sperm cells on the ATH1 platform (Borges et al. 2008). The sperm cell transcriptome of 5829 genes was smaller than that of mature pollen (7177 genes) and showed considerable divergence, with 65.4 % of genes in common (Fig. 4a). Compared with seedlings and pollen, around 2400 transcripts were enriched in sperm, with 642 (11 %) detected only in sperm. Wider comparisons with sporophytic data sets refined a set of 74 sperm-preferential transcripts likely to be sperm cell-specific. While the major GO categories enriched in pollen were membrane transport, signalling, and vesicle trafficking, among sperm cell-preferential transcripts, those showing the most enrichment were DNA repair, ubiquitin proteasome system, and cell cycle, consistent with the propagation and maintenance of germline integrity (Pina et al. 2005; Borges et al. 2008). Compelling evidence for the role of the ubiquitin proteasome system in male germline cell cycle progression includes the role of the SCF^FBL17^ E3 ubiquitin ligase complex (Kim et al. 2008; Gusti et al. 2009) and a pair of Ub-specific proteases (UBP3/UBP4) (Doelling et al. 2007). Small non-coding RNA pathways and DNA methylation pathways were also upregulated in sperm compared with the vegetative cell (Borges et al. 2008). For example, the DNA methyltransferase (MET1) is enriched in sperm, consistent with the active role of MET1 in the maintenance and epigenetic inheritance of CG-context methylation (Saze et al. 2003; Saze 2008; Calarco et al. 2012).

The sperm cell transcriptome has been used to inform the selection of genes of interest for further study. For example, it was used effectively to help distinguish spurious from genuine target genes, following ectopic expression of the male germline-specific MYB transcription factor, DUO1, in seedlings (Borg et al. 2011). Application of a sperm cell filter reduced the number of potential DUO1-activated target genes (DATs), from 124 to 63 genes, including those shown to play a role in gamete fusion (GCS1/HAP2; Mori et al. 2006; von Besser et al. 2006), sperm–egg adhesion (GEX2; Mori et al. 2014), and more recently, germ cell division and sperm cell specification (DAZ1 and DAZ2; Borg et al. 2014).

## The Arabidopsis developmental transcriptome

Developmental resolution to the transcriptome was enabled by the use of density gradient centrifugation to separate four stages from microspore to mature pollen (Honys and Twell 2004): uninucleate microspore (UNM), bicellular pollen (BCP), tricellular pollen (TCP), and mature pollen grain (MPG). In total, 13,977 genes showed male gametophyte expression, with transcriptome size decreasing progressively from 11,565 genes in UNM to 7235 genes in MPG (Fig. 4a). In contrast, the percentage of pollen-specific genes increased from 6.9 % at UNM to 8.6 % at MPG, reflecting the differentiation and functional specialisation of mature pollen. Pairwise comparisons showed expression profiles to be well correlated for UNM and BCP (*R* = 0.96) and for TCP and MPG (*R* = 0.86); however, profiles were less similar for BCP and TCP (*R* = 0.54) stages.

This study was the first to document dynamic changes in gene expression during male gametophyte development and to quantify the numbers of genes contributing to early and late expression programs on a genomic scale (reviewed in Twell et al. 2006). This study was also the first to perform hierarchical cluster analysis, allowing co-regulated genes to be identified. Different co-expressed clusters of genes were found in UNM-BCP compared with TCP-MPG stages, revealing a phase shift in gene expression between BCP and TCP stages. Core cell cycle genes and transcription factors were enriched in UNM-BCP, while genes for signalling and cell wall metabolism were overrepresented in TCP-MPG, consistent with the early proliferative and late differentiation phases of pollen development. Moreover, the transcriptome of cell suspension cultures was more similar to UNM (*R* = 0.47) than to MPG (*R* = 0.13), reflecting the undifferentiated and proliferative state of microspores. Here, it is interesting to note the role of auxin in maintaining dedifferentiation in cell cultures with the peak levels of bioactive auxin, which occurs in microspores (Cecchetti et al. 2008).

The reported profiles of 607 transcription factors (TFs) expressed during pollen development (Honys and Twell 2004) have been exploited in reverse genetic approaches to study regulatory networks (Verelst et al. 2007a; Gibalova et al. 2009; Leydon et al. 2013, Liang et al. 2013; Xia et al. 2014), as well as in the broad phenotypic screening of T-DNA insertions in TF genes (Renak et al. 2012). For example, while the MADS family are collectively under-represented in the pollen transcriptome (Honys and Twell 2004), the non-classical lineages, including the AtMIKC* genes, are overrepresented (Pina et al. 2005). In the first application of these data, Verelst et al. (2007a) explored the function of five MIKC* MADS-box proteins (AGL66, AGL104, AGL67, AGL65, and AGL94), which are co-expressed in late stages of pollen development. MIKC* heterodimer pairs were shown to bind MEF2-type CArG-box motifs, which were found to be highly enriched in the promoters of genes selectively expressed in TCP/MPG during pollen maturation (Verelst et al. 2007a). Moreover, *agl66/104* double mutants showed functional redundancy with severe in vitro germination defects and reduced male transmission. In a further pioneering study, Verelst et al. (2007b) profiled the pollen of double and triple mutants, providing a unique insight into the complexity of the MIKC* TF network that directs a cellular differentiation network during pollen maturation (Table S1). This was further elaborated upon to include phenotypic analysis and profiling of pollen from quadruple mutants, highlighting the large-scale changes in transcripts associated with the absence of MIKC* and supporting the model of Verelst et al. (2007b), whereby the MIKC* network represses genes for early development and activates pollen maturation genes (Adamczyk and Fernandez 2009).

## The Arabidopsis progamic phase pollen transcriptome

In the first study to show transcriptome changes during pollen germination and tube growth of Arabidopsis, Wang et al. (2008) used ATH1 arrays to profile mature (dry) pollen (MP), germinating pollen (GP) cultured in vitro for 45 min, and pollen tubes (PT) after 4 h of in vitro culture. The size of the transcriptome increased from 3945 genes in MP to 4892 genes in PT (Fig. 4a). The percentage of stage-specific genes also increased progressively, from 4.1 % at MP to 14.9 % in PT, with more genes being up- and down-regulated from GP to PT than from MP to GP. Some biological processes enriched in germinating pollen were also overrepresented in pollen tubes, including cell wall metabolism, signalling, and cellular transport. Processes showing the greatest change during pollen germination were stress response and transcription, while in pollen tubes, metabolism and signalling components showed the greatest change. These observations reflect physiological differences, with germinating pollen activated from a quiescent state and pollen tubes actively growing by tip extension.

Communication between pollen and pistil and the stylar environment has important roles in pollen tube growth in vivo (see Taylor and Hepler 1997; Palanivelu and Tsukamoto 2012). For example, it takes around 4 h for the pollen tube to reach the ovule in Arabidopsis (a distance >400 µm), but at 4 h, in vitro-grown pollen tubes are only about 150 µm (Wang et al. 2008). In addition, the growth of pollen tubes in vitro is non-directional and targeting of ovule explants is low, unlike ‘conditioned’ pollen tubes grown through the pistil (Palanivelu and Preuss 2006).

Three studies have examined the transcriptional landscape of Arabidopsis pollen tubes, factoring in pollen tube crosstalk with the pistil and pollen tube guidance (Qin et al. 2009; Chen et al. 2014; Lin et al. 2014).

To examine the role of ‘pistil conditioning’, Qin et al. (2009) compared the transcriptome of semi-in vivo pollen tubes (SIV-PT) grown through stigma–style explants with those of mature pollen and in vitro-grown pollen tubes (PT) at 0.5 h and 4 h. Although similar numbers of genes were expressed in mature pollen and in vitro pollen tubes, SIV-PT expressed a greater number (7044 genes; Fig. 4a). SIV-PT also had the largest number of pollen stage-specific genes (1254), suggesting that growth through the pistil elicits a significant change in the pollen tube transcriptome. Further, a set of 383 genes, uniquely expressed in SIV-PT, was enriched for genes involved in signalling (e.g. transmembrane receptors and protein kinases), defence response (e.g. TIR-NBS-LRR receptors), and cell extension (transporters and antiporters). Reverse genetic analysis of 33 genes induced during pollen tube growth identified five required for optimal tube growth in vitro, while mutations in two further genes showed pollen tube navigation defects in vivo. One of the in vivo effect genes, At3g18000 (*XIPOTL*), was specifically up-regulated in SIV-PT and is required for phosphatidylcholine synthesis (Cruz-Ramirez et al. 2004), implicating lipid signalling and/or plasma membrane composition in pollen tube growth though the pistil.

Chen et al. (2014) extended the semi-in vivo approach to factor in the role of diffusible pollen tube attractants and monitored the transcriptome of semi-in vivo-guided pollen tubes (SIV-PG) by positioning ovules below pollen tubes emerging from pistil explants. Using a 12 × 135 K Arabidopsis gene chip (Roche-NimbleGen), 719 genes were identified to be specifically expressed in SIV-PG. Gene families, such as defensin-like (DEFL), leucine-rich repeat receptor like kinases (LRR-RLKs), and TIR-NBS-LRR receptors, were specifically enriched in SIV-PG, but surprisingly there was little overlap with candidate pollen tube guidance genes from the SIV-PT system of Qin et al. (2009). Phenotypic screening of 18 confirmed T-DNA insertion lines for pollen tube guidance defects was unsuccessful, highlighting potential redundancy among the often large, gene families involved. However, in a dominant negative approach involving the expression of a ‘kinase-deleted’ variant, phenotypes consistent with pollen tube guidance defects were observed for one LRR-RLK gene (Chen et al. 2014).

Lin et al. (2014) used a novel application of the ‘TRAP’ method (translating ribosomes affinity purification) to the male gametophyte, allowing in vivo gene expression data to be gathered for the progamic phase. This approach involves immunopurification (IP) of ribosome-associated mRNA to identify transcripts undergoing active translation—the ‘translatome’ (Zanetti et al. 2005). Transgenic plants in which epitope-tagged ribosomal protein L18 was expressed from the pollen-specific LAT52 promoter were used for mRNA–ribosome complex isolation from unpollinated buds (IP-bud), open flowers (IP-in vivo), as well as 4.5 h in vitro-grown pollen tubes (IP-in vitro). The LAT52 promoter is active in the late microspores and after PMI in the vegetative cell of Arabidopsis and is expected to capture the vegetative cell translatome (Eady et al. 1994; Grant-Downton et al. 2013), In IP-bud samples, 8140 genes had corresponding transcripts undergoing translation with a similar number in IP-in vivo samples, but significantly fewer (5188 genes) than in the IP-in vitro pollen tubes. In the IP-in vivo data set, 519 genes were specifically enriched, implicating these genes in the growth and guidance of the pollen tube in the gynoecium and in response to fertilisation. This set of genes was enriched for molecular functions associated with heme, which has been implicated in sperm cell discharge through pollen tube rupture (Lin et al. 2014). Whereas an overlap of 13 % (67/519) was observed between IP-in vivo-enriched and pollination-induced mRNAs (Boavida et al. 2011), there was only 4 % (17/383) overlap between semi-in vivo and IP-in vivo, pollination-induced mRNAs, indicating a significant distinction between enriched transcripts from semi-in vivo and true in vivo pollen tubes. Reverse genetic analysis identified mutations in three IP-in vivo-enriched genes, *iv2* (methyl esterase 8, MES8), *iv4 (*glutathione s-transferase, GSTU26), and *iv6* (xyloglucan endotransglucosylase/hydrolase 19), which showed reduced male transmission. These mutants showed defective micropylar guidance, pollen tube burst, and ovules receiving multiple pollen tubes (polytubey).

**Fig. 5:**
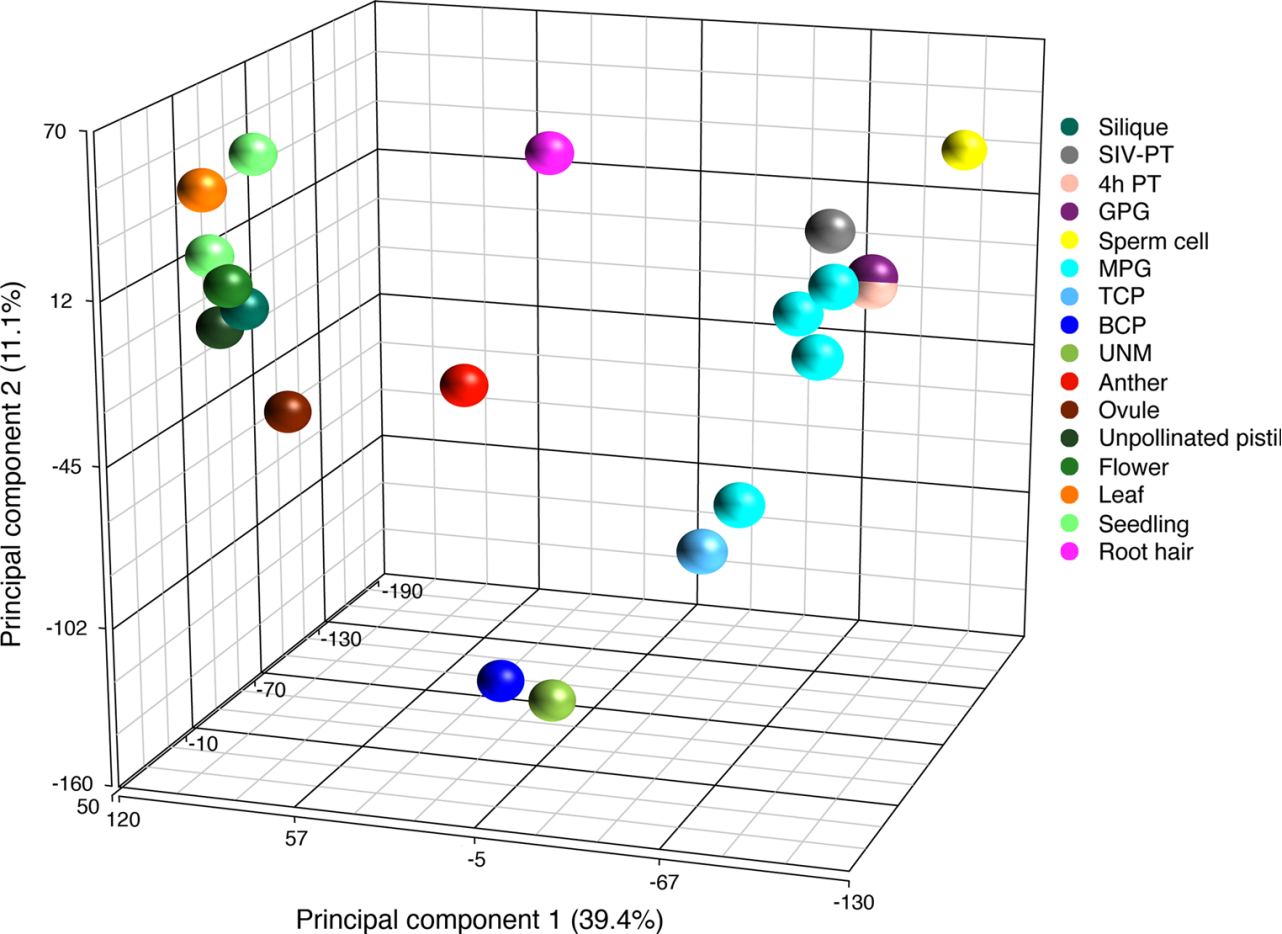
Principal component analysis (PCA) of ATH1 microarray data for male gametophyte and selected sporophytic samples. The data sets included are from the following publications: Honys and Twell (2004), Borges et al. (2008), Qin et al. (2009), Phan et al. (2011), Becker et al. (2014). PCA was generated using RMA-normalised data in Partek^®^ Genomics Suite^®^ (Partek Inc., Missouri)

Collectively, the transcriptome analyses outlined above establish some unifying features of Arabidopsis pollen development. First, mature pollen grains possess the most unique transcriptome with the least transcript diversity when compared with the sporophyte. Second, the transition from proliferating microspores to differentiated pollen is associated with large-scale repression and de novo transcription of an increased number of pollen-specific genes. Third, sperm cells possess a similarly reduced, but diverse and unique transcriptome. Fourth, the germination and directional growth of the pollen tubes involves stored transcripts and de novo transcriptional responses associated with signalling and communication with the gynoecium. The distinctive transcriptomes associated with the different phases of pollen development and the germline are contrasted with those of the sporophyte in the principal component analysis shown in Fig. 5.

## Comparative pollen transcriptomics

The analysis of pollen transcriptome profiles for diverse angiosperms enables the conservation of the molecular mechanisms underlying the different phases of pollen development to be explored. Pollen microarray and/or RNA-seq data are available for at least 10 different species including Arabidopsis. Lists of the current data sets are compiled in Tables S1 and S2. Transcriptome profiling at multiple points in development has only been reported for Arabidopsis, cultivated rice, and tobacco (see Table 1), which differ in specific features, such as the timing of germ cell division and length of the progamic phase.

### Rice

The transcriptome of developing rice pollen has been investigated in two independent studies, the first using laser microdissection of tapetum, microspores, and pollen, and the 44-K rice oligo microarray platform (Suwabe et al. 2008; Hobo et al. 2008). K-means cluster analysis of the data revealed eight gene clusters with synchronous gene expression in tapetum and microspores involving a total of 10,810 genes. Within these clusters, there was enrichment for transcription, secondary metabolism (e.g. chalcone synthase, chalcone reductase, and phytoene synthase), and lipid synthesis and metabolism processes associated with construction of the pollen wall. These observations counter the view of the tapetum simply as a nurse cell layer, recognising shared pathways and the common developmental origins of tapetal cells and microspores (Scott et al. 2004; Hobo et al. 2008).

A subsequent study using the 57 K Affymetrix Rice Genome Array (Wei et al. 2010) showed a progressive decrease in the number of expressed genes, from 14,590 genes in uninucleate microspores to 5939 genes in mature pollen and a similar number (5945 genes) in germinated pollen (Fig. 4b). Similar to Arabidopsis, the rice pollen transcriptome was highly reduced compared to the sporophyte, where 17,383 genes were detected in roots and 17,242 in leaves. The overall patterns of genes expressed mirrored observations from Arabidopsis, with highly correlated early (UNM and BCP; *r* = 0.82)- and late (TCP and MPG; *r* = 0.76)-stage profiles and a distinct phase shift between. The authors described a ‘U-type’ change for pollen-preferential or stage-specific transcripts in rice and Arabidopsis, with the least number of genes preferentially expressed at bicellular stage. This trend reflects the bicellular stage as transitional between early proliferative and late differentiation phases. Compared with Arabidopsis, rice showed a steeper decline in the number of genes expressed between TCP and MPG stages, suggesting differences in the onset of large-scale transcriptional repression and/or in the rates of transcript turnover (Fig. 4).

Despite overall similarities, significant differences were noted in the distribution of GO categories for genes expressed at different stages between rice and Arabidopsis. For example, rice expressed more stage-enriched transcripts associated with defence and stress response in mature pollen, while rice pollen was more enriched for signalling at bicellular stage. Overall comparisons revealed that 62.4 % (1195 genes) of stage-enriched genes from rice had homologues in the Arabidopsis genome; however, only 56.6 % (677 genes) of these were expressed, with a very small proportion, 3.2 % (22 genes), being preferentially expressed in Arabidopsis pollen. Moreover, the analysis of regulatory proteins identified many TF classes, which were shared between rice and Arabidopsis, but the expression of a set of unique TFs in rice pollen indicates that regulatory networks will show significant differences (Wei et al. 2010).

Direct evidence for the conservation of an important TF network between rice and Arabidopsis includes a study of the MIKC* MADS-box network, which is required for pollen maturation (Liu et al. 2013). While Arabidopsis encodes five pollen-expressed MIKC* genes, the rice genome has three genes divided between S- (OsMADS62, OsMADS63) and P- (OsMADS68) clades, which show conserved expression in pollen. Similar to observations in Arabidopsis, mutants affecting rice P- or S-clade MIKC* genes displayed defects in pollen germination in vitro, but also showed reduced pollen viability and abnormal starch accumulation (Liu et al. 2013). Moreover, the transcriptome data of Wei et al. (2010) were used to show that the promoters of genes expressed during late pollen development were enriched for MIKC* binding sites. This work indicates that the function of the MIKC* regulatory network in pollen development has been conserved, since the divergence of eudicots and monocots, some 150 million years ago (Liu et al. 2013).

### Tobacco

Transcriptome analysis of the male gametophyte from tobacco using the Agilent 44-K tobacco microarray, originally consisting of three stages (mature pollen, 4, and 24 h in vitro pollen tubes; Hafidh et al. 2012a, b), was recently extended to include earlier stages of development (uninucleate microspores, early bicellular pollen, and late bicellular pollen; Bokvaj et al. 2015). Similar to the observations in Arabidopsis and rice (Fig. 4), transcriptome complexity was reduced during pollen maturation in tobacco, although the onset of the decline was later, with an unusually low diversity of transcripts (16,700) detected in microspores relative to early and late bicellular pollen (22,872 and 19,750, respectively). Although some features, such as the abundance of translational components at early stages and the overrepresentation of transport and cytoskeleton at late stages of pollen development (Bokvaj et al. 2015), reflected patterns observed in Arabidopsis and rice, the temporal shift in maximum transcriptome complexity from microspore to the bicellular stage may reflect different demands on the rate of development, and it will be interesting to examine whether this is a general feature of species that shed bicellular pollen. Division of the germ cell in tobacco occurs 8–12 h after pollen germination, such that 4-h pollen tubes harbour M-phase generative cells, while 24-h pollen tubes contain sperm. During tobacco pollen tube growth, a moderate increase in the number of detectable transcripts was observed, from 13,966 in mature pollen to 14,420 in 24-h pollen tubes, with 3597 transcripts in common between these three stages. Transcripts of 699 genes (4.8 %) accumulated significantly (>fivefold) compared to mature pollen and the 4-h pollen tube, while 320 genes (2.2 %) accumulated de novo after 4 h, highlighting the extended transcriptional capacity of pollen tubes cultivated in vitro (Hafidh et al. 2012a, b). The delay in germ-cell-cycle progression, until 10–12 h of in vitro pollen tube growth, was linked with the presence of transcripts for cell cycle inhibitors, such as RBR1 and DEL3. Transcripts for the G2/M-phase activator CYCB1;1 peaked during the transition to PMII, together with the expression of the tobacco orthologue of *AtDUO1* (*NtDUO1*; B25, Kyo et al. 2003), which is required for germline CYCB1;1 accumulation in Arabidopsis (Brownfield et al. 2009a). Although the expression of cell cycle repressors and activators requires validation in tobacco germ cells, these data support the conservation of DUO1-mediated mitotic progression and differentiation between Arabidopsis and tobacco.

### Other species

Other species for which mature pollen transcriptome data have been published include maize (Ma et al. 2008; Davidson et al. 2011; Chettoor et al. 2014), soybean (Haerizadeh et al. 2009), grapevine (Fasoli et al. 2012), potato (Sanetomo and Hosaka 2013), woodland strawberry (Hollender et al. 2014), and most recently lily (Lang et al. 2015).

The maize pollen transcriptome has been analysed in three independent studies focussed on reproductive development. In the microarray study of Ma et al. (2008), 10,545 different transcripts were detected in maize pollen, which was extended in the two RNA-seq experiments to 13,418 genes (Davidson et al. 2011) and 14,591 genes (Chettoor et al. 2014). The maize pollen transcriptome was typically reduced in complexity, expressing, for example, only 57 % of the mean number of genes expressed in 12 other sporophytic tissues (Davidson et al. 2011). Moreover, pollen was also among tissues with the most extreme range of transcript levels, showing the highest maximum expression levels (Davidson et al. 2011). The classification of array data (Ma et al. 2008) into high (237 transcripts) and medium (5547) abundance classes was also strikingly congruent with the original estimates (240 and 6000, respectively) for maize pollen based on R_0_t curves (Willing et al. 1988). However, the low copy number, slow to hybridise transcript class, appears to be substantially overestimated in R_0_t hybridisations (Ma et al. 2008). Comparative analysis by Ma et al. (2008) indicated significant conservation in gene expression programs between Arabidopsis and maize pollen. Of 4407 homologues of Arabidopsis pollen-expressed genes represented on the maize array, 3444 (78 %) were expressed in maize pollen and highly expressed genes in each species showed substantial overlap.

Since maize is an allotetraploid consisting of two subgenomes, the relative contributions of each subgenome to the trancriptome can be determined to explore potential bias between the gametophyte and sporophyte. Previous studies revealed that subgenome 2 is characterised by reduced expression and a higher rate of gene loss, relative to subgenome 1 (Schnable et al. 2011). However, in their study, Chettoor et al. (2014) discovered, that the pollen transcriptome was overrepresented for subgenome 2, compared to the other tissues assessed. The basis for this difference was linked to the retention of a greater proportion of duplicate gene pairs derived from both subgenomes for pollen, with both members of duplicate pairs also more likely to be pollen-enriched. This suggests that selective pressure could be acting on pollen to maintain functional copies of both homeologues following tetraploidisation, due to increased sensitivity of pollen to gene dosage effects (Chettoor et al. 2014).

The soybean *(Glycine max*) pollen transcriptome is the first described for a legume and for a bicellular pollen species (Haerizadeh et al. 2009). Soybean pollen expressed 10,299 transcripts representing only 37 % of those detected in sporophytic tissues, while 7.9 % were pollen-specific. The most abundant transcripts were enriched for cell wall-modifying enzymes, signalling genes, and transporters, typical for Arabidopsis pollen. There was divergence, however, in the number and diversity of regulatory proteins, and small RNA pathway proteins were underrepresented. Heat-shock proteins and heat-shock TFs were up-regulated in mature soybean pollen, in contrast to Arabidopsis, in which they were up-regulated specifically in germinating pollen and proposed to act as molecular chaperones to accommodate the intense physiological activities associated with pollen germination and tube growth (Wang et al. 2008).

Pollen microarray data for grapevine (*Vitis vinifera*) were generated as part of a gene expression atlas for this species (Fasoli et al. 2012). Similar to other studies, mature pollen showed a distinctive transcriptome including enrichment for cell wall-modifying enzymes. Although reduced in complexity compared to some sporophytic samples, in contrast to previous studies, the pollen transcriptome was not among the least complex, with berries, tendrils, and stems showing lower complexity, perhaps reflecting the specialised activities of some tissues and the diversity of tissue samples analysed.

The pollen transcriptome of woodland strawberry (*Fragaria vesca*) was generated by as part of a study to develop *F. vesca* into a model plant for the Rosaceae (Shulaev et al. 2011). The transcriptomes of mature pollen and microspores isolated by laser capture microdissection were determined by RNA-seq (Hollender et al. 2014). Similar to studies in Arabidopsis and rice, *F. vesca *mature pollen and microspores showed distinctive transcriptomes, with mature pollen showing the least complexity (11,540 genes) compared with microspores (33,109 genes) or all floral tissues combined (34,527 genes).

Lily (*Lilium longiflorum*) provides an established model for pollen germination and tube growth, with significant advantages for physiological and biochemical analyses; however, only limited molecular data are available. The recent analysis by Lang et al. (2015), involving the RNA-seq of a normalised cDNA library (pooled from dry, hydrated, germinating pollen and pollen tubes), provides the first comprehensive overview of the lily pollen transcriptome. Assembled transcripts revealed conserved features when visualised with MAPMAN software tools and compared with RNAseq data from Arabidopsis pollen (Loraine et al. 2013). This study provides useful data sets and tools to search for lily sequences of interest enabling comparative studies.

## Comparative male germline data

Exploring gene expression in the male germline has benefitted from high-throughput screens using EST libraries. This approach overturned previous notions of transcriptionally inert male gametes and delivered valuable large-scale data for generative cells of lily (Okada et al. 2006), and for sperm cells of maize (Engel et al. 2003), *Plumbago* (Gou et al. 2009), and tobacco (Xin et al. 2011). The comparative analysis of maize sperm EST data identified the conserved gamete-expressed genes, *GEX1* and *GEX2* in Arabidopsis (Engel et al. 2005), which have important roles and use as research tools in gamete biology (Brownfield et al. 2009a, b; Alandete-Saez et al. 2011; Mori et al. 2014).

The in-depth profiling of purified sperm cells from a crop species was first achieved for rice using the 57-K microarray (Russell et al. 2012). Strikingly, rice sperm cells expressed 10,732 genes, twice as many as sperm cells from Arabidopsis. Rice sperm cells were also found to express more genes than mature pollen (8101 genes), in contrast to Arabidopsis sperm, which express fewer than in mature pollen (Fig. 4). Similar to Arabidopsis, rice sperm cells were enriched for genes associated with ubiquitin pathways, DNA modification and repair, and chromatin remodelling. The enrichment of cell cycle genes observed in Arabidopsis sperm (Borges et al. 2008), however, was not reported in rice, consistent with the arrest of Poaceae sperm in G1 (Friedman 1999). The expression of more than 70 sperm-enriched TFs was reported in rice, and homologues of several genes in the Arabidopsis DUO1 regulatory network were also selectively expressed, including OsDUO1 (although not present on the array), supporting the conservation of a key germline regulatory network (Russell et al. 2012).

A simple cell isolation procedure involving the fractionation of sperm cells from burst pollen grains has allowed the sperm cell transcriptome to be compared with that of sperm cell-depleted vegetative cells (Anderson et al. 2013). The number of genes reliably detected was 16,985 in sperm and 18,611 in vegetative cells, which reflects the reduced complexity of sperm compared with mature pollen of Arabidopsis (Borges et al. 2008). This is in contrast to the findings in rice using the 57-K microarray, where more genes were detected in sperm than in mature pollen (Russell et al. 2012). The top 50 transcripts in sperm showed a broader range of expression with higher peak levels than those of the vegetative cell, indicating that the reduced size and transcriptional output of sperm cells do not limit the capacity of sperm to express individual genes (i.e. ATPase) at very high levels. Furthermore, the overlap between the vegetative and sperm cells was found to be much higher (by around 25 %) than between either of these and the egg cell, reflecting the common lineage of the sperm cells and vegetative cell.

In both studies of the rice sperm cell transcriptome, diversity in the complement of histones was noted, particularly type H2B and H3, with enrichment for at least one member of each of the five major histone types (Russell et al. 2012). All three homologues of HTR10 (MGH3), the Arabidopsis male germline-specific histone H3.3, are expressed in rice sperm cells, together with histone-modifying proteins. Histone modification genes were differentially expressed between the vegetative cell and sperm cells (Anderson et al. 2013). For the JUMONJI (JMJ) histone demethylase family, which uses histone H3 as a substrate and is involved in transcriptional repression, vegetative cells lacked most of the H3K36-modifying JMJ proteins expressed in sperm cells, with sperm cells expressing the H3K27 demethylase ZOS1-20 to higher levels than in the vegetative cell. In common with Arabidopsis, rice sperm cells express MET1, while transcript levels are very low in vegetative cells, consistent with the maintenance of CG methylation in the male germline and its absence in the vegetative cell (Calarco et al. 2012). Equally, DRM2 is expressed in the vegetative cell implying that RNA-directed CHH methylation is conserved. However, unlike Arabidopsis, components of the RNA-directed DNA methylation (RdDM) machinery are expressed in rice sperm, including DCL3, DRM2, and RDR2, although the latter is only reported by Anderson et al. (2013). Interestingly, a similar number of sequence reads was found to map to transposon-associated repeats in vegetative cells and in the sperm cells, and previous reports have described a high proportion (8 % of transcripts) of retroposon sequences in maize sperm (Turcich and Mascarenhas 1994; Engel et al. 2003). These findings indicate that differences may exist between rice and Arabidopsis in the mode of epigenetic regulation of siRNA-mediated gene silencing in the male germline.

## Integration and analysis of pollen transcriptome data

With the wealth of transcriptome data now available for the male gametophyte, meta-analyses can be used to identify genes with common expression and activities between multiple data sets. The data can be used to construct in silico-derived co-expression networks and are based on the assumption that genes with similar expression patterns are likely to interact with each other. In a pioneering study in rice, Aya et al. (2011) adopted a co-expression analysis to identify ‘meiosis’ and ‘pollen wall synthesis’ sub-networks. The study analysed 176 microarray data sets, including LM-microarray data separating tapetum and male gametophyte cells, to identify a robust pollen wall synthesis co-expression network, enriched for sporopollenin-associated genes together with genes not previously implicated in exine formation. This study demonstrates the value of co-expression network analysis and how the separate transcriptomes of pollen and tapetum cells can increase the precision and resolving power of network construction.

Integrative approaches utilising data within and between species can also be used to explore common cellular processes in different cell types. Two independent studies have searched for a common set of genes associated with polar cell growth. As part of a study of the tobacco pollen tube transcriptome, Hafidh et al. (2012a) incorporated cross-species comparisons, including transcriptome and proteome data to identify Arabidopsis homologues of genes co-expressed in tobacco pollen tubes and roots (3264 genes), of which 78 genes overlapped with genes known to possess root hair-specific promoter motifs (Won et al. 2009). This subset, extended to a candidate list of 104 genes, showed enrichment for vesicle transport, signal transduction, translation, and cytoskeleton. Evidence for the function of three candidate ‘root hair–pollen tube’ genes in the progamic phase was shown, by transfection of antisense RNA, resulting in reduced in vitro pollen tube growth. In a different strategy, Becker et al. (2014) generated purified root hair and pollen transcriptomes in Arabidopsis, incorporating a distinction between apical (polar) and diffuse (non-polar) cell growth to identify a molecular signature for polar cell growth. This identified 4989 genes co-expressed in root hairs and pollen, with 277 showing enrichment compared to diffuse growth cell types and 105, which were exclusive to root hairs and pollen. An interesting finding based on promoter analysis of a subset of 49 co-expressed apical cell growth-specific genes was the identification of a sequence motif enriched in hypoxia-induced genes, supporting the hypothesis that tip growing cells experience anaerobic conditions (Becker et al. 2014). Despite the different strategies employed in the two studies, there was notable overlap, with 48 out of the 104 candidate polar cell expansion genes of Hafidh et al. (2012a) detected in Arabidopsis root hairs and pollen, but only three of these genes were found to be enriched compared with cell types showing non-polar growth (Becker et al. 2014).

Computational approaches involving meta-analyses have also been used to construct and explore a regulatory network of TF activity during pollen development (Wang et al. 2011). Network component analysis (NCA) was applied to a total of 23 data sets encompassing developmental and progamic phases to construct a regulatory network involving 19 TFs, 101 target genes, and 319 regulatory interactions. Although NCA can infer hidden TF activities by taking advantages of the prior knowledge of network structure, most of the regulatory information and regulatory pairs retrieved from co-expression analysis remain hypothetical. Nevertheless, NCA did include the pollen-specific factor WRKY34 (Honys et al. 2006), which is negatively regulated by MIKC* transcription factors and involved in cold sensitivity and pollen maturation (Verelst et al. 2007b; Zou et al. 2010; Guan et al. 2014). Currently, none of the network predictions including TF interactions and target genes are reflected by experimental data, and further effort is required to gather empirical knowledge of system components and to validate and improve network models.

Most recently, pollen transcriptome data have been used in two phylostratigraphic studies, to address a novel hypothesis in plants concerning the origin of ‘new’ or ‘orphan’ genes. In animals, new genes, which provide material for evolutionary innovation, show testes-biased expression and are thus regarded as the birthplace for new genes in an “out of testes” hypothesis (Kaessmann 2010). Wu et al. (2014) propose a corresponding “out of pollen” hypothesis and Cui et al. (2015) a “young genes out of the male” hypothesis for the origin of new genes in flowering plants. In the studies of Wu et al. (2014) in Arabidopsis and Cui et al. (2015), which included Arabidopsis and rice, the phylogenetically youngest transcriptome was found in pollen, based on the analysis of transcriptome age index (TAI; Domazet-Loso and Tautz 2010). Moreover, the sperm transcriptomes of Arabidopsis and rice were shown to be similarly young (Cui et al. 2015). Both studies highlight the enrichment of TEs in the new genes and the importance of epigenetics and chromatin states in the vegetative cell and/or the germ cells in shaping transcriptome age.

## Concluding remarks and outlook

Transcriptome studies of the male gametophyte have not only increased knowledge and understanding, but also improved the efficacy of experimental strategies: first, in describing transcript profiles (throughout development and in specific cell types); second, by informing experimental design (such as by gene selection for reverse genetics), through query-based and co-expression analysis; and third, by providing the raw materials to build gene networks and a methodology to understand how these are affected in pollen mutants. All three approaches have been exploited (to a greater of lesser extent) by the Arabidopsis community, and progress encompassing all three approaches in other model and non-model species is expected as more data are accumulate, together with improved methods for exploring gene function, such as genome editing (Belhaj et al. 2013).

Microarrays have been the dominant profiling technology, providing a uniform platform from which to explore the expression of individual genes and gene families, to build co-expression networks and to aid the design of reverse genetics experiments. For multiple reasons, including reduced costs of replication, higher sensitivity, and information content, RNA-seq is set to become the dominant profiling technology in future studies (Liu et al. 2014). Since RNA-seq requires little a priori knowledge of the genome, this enables transcriptome-based studies of non-model species, such as *Capsella grandiflora,* in which pollen-specific genes were identified to show stronger purifying selection and higher rates of positive selection than sporophytic genes (Arunkumar et al. 2013). RNA-seq has also opened the way for the small RNA landscape of pollen to be explored (Grant-Downton et al. 2009; Slotkin et al. 2009; Zhang et al. 2009; Borges et al. 2011; Li et al. 2013). By integration of mRNA transcriptome data and other genome-wide information, such as bisulphite sequencing, our understanding of the epigenomic landscape of the gametophyte and male germline has also advanced (Calarco et al. 2012). Pollen transcriptomics can also be applied more widely to develop tools important to the biotechnology sector (Oo et al. 2014) and to address agronomically important questions such as tolerance to environmental stresses linked to global climate change. For example, pollen heat stress can cause sterility and associated crop losses, and efforts are underway to explore the molecular networks associated with thermotolerance in a broader system-level analysis (Bokszczanin et al. 2013; Giorno et al. 2013; Loraine et al. 2015).

### **Author contribution statement**

NR and DT wrote the manuscript. Both authors read and approved the manuscript.

## Electronic supplementary material

Below is the link to the electronic supplementary material.

Supplementary material 1 (XLSX 16 kb)

Supplementary material 2 (XLSX 15 kb)
